# Describing the outcomes and factors of using pathogen genomic results in public health

**DOI:** 10.3389/fpubh.2026.1904109

**Published:** 2026-08-17

**Authors:** James D. H. Ong, Tehzeeb Zulfiqar, Daisy Wang, Stephanie Panchision, Aaliya Ibrahim, Kathryn Glass, Martyn D. Kirk, Brad Astbury, Angeline Ferdinand

**Affiliations:** 1 Centre for Pathogen Genomics, Department of Microbiology and Immunology, The Peter Doherty Institute for Infection and Immunity, The University of Melbourne, Melbourne, VIC, Australia; 2 National Centre for Epidemiology and Population Health, College of Law, Governance and Policy, Australian National University, Canberra, ACT, Australia; 3 Evaluation and Implementation Science Unit, Centre for Health Policy, Melbourne School of Population and Global Health, The University of Melbourne, Melbourne, VIC, Australia

**Keywords:** antimicrobial resistance, case studies, implementation science, infectious disease, pathogen genomics, pathogens, qualitative research

## Abstract

**Background:**

Although many countries are incorporating pathogen genomics into their public health systems, previous studies rarely examine their implementation, use and outcomes. This study describes how pathogen genomics is implemented and used in public health to guide surveillance and outbreak responses against infectious disease.

**Methods:**

We examined 11 case studies of pathogen genomics use in surveillance programs and outbreak responses against infectious disease across Australia and New Zealand. Information on each case study was collected via document review and key informant interviews and summarized in case reports. Case reports were then compared to identify similarities and differences in outcomes and factors.

**Results:**

Although pathogen genomics served various functions in supporting public health investigations and responses against infectious disease, their impact varied. This ranged from providing information about a clinical case or public health response to informing changes in public health investigations and responses against infectious disease. Timeliness and novelty were two factors that appeared to distinguish the impact of pathogen genomics in public health among case studies. Other factors such as end users’ literacy and attitudes were similar across case studies, but may help highlight the need for consistent processes to support implementation of pathogen genomics in public health.

**Conclusion:**

This study describes how pathogen genomics is being implemented across different pathogens and contexts in public health. These results will inform future research and implementation programs that aim to improve public health pathogen genomics programs. This may lead to enhanced use and outcomes to facilitate more tailored, timely and effective public health responses against infectious disease.

## 1 Introduction

Clinical, epidemiological and laboratory data inform public health surveillance, investigations and responses against infectious disease and antimicrobial resistance (AMR) (1). From the 2010s, pathogen genomics is being increasingly used to monitor and respond to infectious disease. Pathogen genomics involves whole genome sequencing to sequence numerous fragments of pathogen DNA and bioinformatics to assemble the fragments into a complete genomic sequence (2–4). Genomic sequences of different isolates from the same pathogen can then be compared to identify single nucleotide polymorphisms that can infer likely infection sources and transmission routes (4). These findings can guide public health investigations and responses against infectious disease (2, 5, 6) and AMR (7–9).

Countries are steadily incorporating pathogen genomics into their infectious disease surveillance systems and outbreak investigations and responses. Initiatives such as the US Food and Drug Administration’s GenomeTrakr program (10, 11) and Public Health England’s Gastrointestinal Bacterial Reference Unit (12) sequence foodborne pathogens and share genomic data to identify foodborne-related outbreaks nationally and globally. During the COVID-19 pandemic, pathogen genomics was used to identify SARS-CoV-2 variants (13), detect SARS-CoV-2 genetic material in wastewater samples (14), and trace the origins of COVID-19 clusters (15–17). Consequently, the COVID-19 pandemic raised the importance of pathogen genomics in contributing to public health responses against infectious disease, highlighted in the World Health Organization’s *Global Genomic Surveillance Strategy for Pathogens with Pandemic and Epidemic Potential, 2022–2032* (18). In Australia, the Australian Pathogen Genomics (AusPathoGen) program was established to incorporate pathogen genomics into state and provincial public health systems across Australia and New Zealand, with the aim to improve surveillance and outbreak responses against infectious disease at the state, territory, provincial and national levels (19).

Public health pathogen genomic programs consist of several processes that can be examined either separately or together. According to the Pathogen Genomics in Public HeAlth Surveillance Evaluation (PG-PHASE) Framework (20), these processes can be split into three groups:

1. The pre-analysis and analysis phase, where isolates are collected and sequenced;
2. The reporting and communication phase, where pathogen genomic results are communicated to end users; and
3. The implementation in public health practice phase, where pathogen genomic results are used to guide public health responses against infectious disease.

Although an evaluation framework exists for public health pathogen genomic programs, their implementation, utility and cost-effectiveness are seldomly evaluated (7). Additionally, no study has looked at how end users (such as policymakers and public health units) use pathogen genomic results to inform public health surveillance and responses against infectious disease. Lastly, despite various factors that could affect how pathogen genomic results are used such as turnaround times to deliver results (7, 21) and end users’ attitudes and knowledge towards pathogen genomics (22, 23), these have not been examined systematically in the same study.

Hence, this study aims to describe the factors and outcomes of using pathogen genomic results to guide surveillance and outbreak responses against infectious disease. This was achieved by examining case studies in Australia and New Zealand where pathogen genomic results were available to inform public health investigations and responses against infectious disease. Case studies were then compared via multiple case study design in terms of what pathogen genomics had achieved and the factors influencing their use and outcomes. Together, this study describes how pathogen genomics is implemented and used in public health, providing insights into how public health pathogen genomic programs could be improved.

## 2 Materials and methods

### 2.1 Study setting and design

The AusPathoGen program is a government-funded research program that aims to incorporate pathogen genomics into public health systems across Australia and New Zealand (19). The AusPathoGen program has three aims:

1. Develop effective genomics-informed responses against infectious disease;
2. Optimize AusTrakka, a pathogen genomic data sharing and analysis platform for Australian and New Zealand public health laboratories (24); and
3. Evaluate the utility and cost-effectiveness of public health pathogen genomic responses in Australia and New Zealand (25).

This study was part of the third aim of the AusPathoGen program. For this study, multiple case study design was used. This involved examining individual case studies of pathogen genomics in Australia and New Zealand to describe its implementation, use and outcomes in public health. Case studies were then compared to identify similarities and differences (26, 27). Multiple case study design was used as it preserves the context of each case study, something that variable-based methods such as regression analysis omit. This enables the outcome and factors to be explained more generally, with examples from specific case studies used to support each statement (28). Additionally, given that little is known on what may affect implementation of pathogen genomics in public health, case studies can be used to systematically explore associations between specific implementation factors and the use of pathogen genomic results.

In the context of this study, multiple case study design had five steps:

1. Outcome and factor definition: The outcome and factors of the use of pathogen genomic results were defined for the study.
2. Case selection: Case studies of pathogen genomics use across Australia and New Zealand were selected for the study.
3. Data collection: Information about each case study was collected via document review and key informant interviews.
4. Within-case analysis: Individual case studies were coded and summarized in case reports.
5. Cross-case analysis: Case reports of different case studies were compared to identify similarities and differences in the outcomes and factors.

### 2.2 Outcome and factor definition

In this study, the outcome was how much end users utilized pathogen genomic results, and how much that use influenced surveillance or outbreak responses against infectious disease. Conversely, factors described potential conditions that may affect the implementation, use and outcomes of pathogen genomic results such as whether pathogen genomic results were received on time (6, 21) and how pathogen genomic reports were formatted (29, 30).

**Figure 1:**
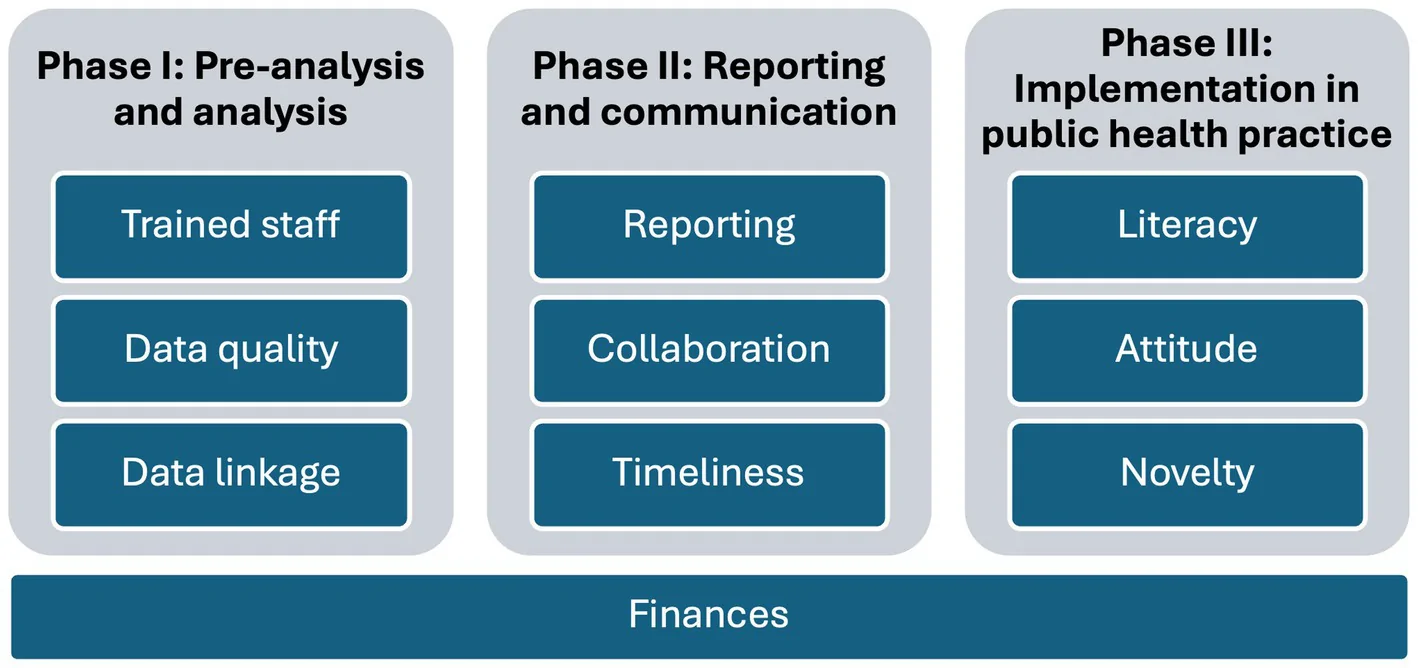
Factors investigated in the study, mapped to a specific PG-PHASE phase.

Factors influencing pathogen genomics implementation, use and outcomes were initially derived by reviewing the PG-PHASE Framework (20) and further consolidated and refined by reviewing peer-reviewed literature and examining case studies. Figure 1 lists the factors that were investigated in the study, along with the PG-PHASE phase each factor belonged to. Table 1 defines the questions that were examined for each outcome and factor.

**Table 1:** The outcome and factors examined in the study, along with their questions.

PG-PHASE phase	Factor	Question
Outcome		How was pathogen genomics used in the case study, and to what extent did it affect public health investigations and responses against infectious disease or AMR?
Phase I: Pre-analysis and analysis	Trained staff	How many technical staff were trained in sequencing and analyzing pathogen genomic data?
	Data quality	How reliable was pathogen genomic data in informing public health responses against infectious disease?
	Data linkage	How easy was it to link pathogen genomic data to laboratory and epidemiological data?
Phase II: Reporting and communication	Reporting	How easy was it for end users to understand pathogen genomic reports?
	Collaboration	To what extent did pathogen genomic experts collaborate with other public health experts and end users to incorporate pathogen genomic data into public health responses?
	Timeliness	How quickly could pathogen genomic results be delivered to end users to inform public health responses against infectious disease?
Phase III: Implementation in public health practice	Literacy	How much did end users understand how to use pathogen genomic data to inform public health responses?
	Attitude	What did end users think about the role of pathogen genomics to inform public health responses against infectious disease?
	Novelty	How much new information did pathogen genomics reveal to end users to inform public health responses against infectious disease?
Finances		To what extent was the government funding pathogen genomic services?

### 2.3 Case selection

Case studies referred to surveillance programs or outbreak responses against infectious disease where pathogen genomics was involved. They were selected based on the following selection criteria:

1. Pathogen genomics was used in a surveillance program or outbreak response against an infectious disease in Australia and New Zealand in 2016 or beyond as this was when pathogen genomics capacity and use in public health within the region started to increase;
2. Most clinical and/or environmental samples of the pathogen were prospectively extracted and sequenced, not solely relying on retrospective genomic data that had already been sequenced, to track how pathogen genomics was prospectively used in public health;
3. There was an attempt to inform end users about pathogen genomic results for the purposes of disease prevention and control, to exclude any case studies that were solely research-based; and
4. The case study did not involve investigations of SARS-CoV-2 as previous studies have already looked at the implementation of pathogen genomics in public health against COVID-19 (15–17).

Case studies were identified via consultation with coauthors and public health staff across Australia and New Zealand, as well as a literature search of peer-reviewed literature over the PubMed, EMBASE and CINAHL databases. If a case study that fulfilled the selection criteria was found, we met with the key contact person to learn more about the case study and to assess whether it could be researched. Data collection only started once the contact person gave permission for research to be conducted on the case study.

### 2.4 Data collection

For each case study, data were collected via document review and key informant interviews.

Document review provided background information on the case study. Peer-reviewed articles, as well as publicly available documents such as government reports, were obtained via Google search. Internal documents that were not publicly available such as genomic and epidemiology reports were obtained in consultation with key informants. All documents were stored in a secure SharePoint within University of Melbourne servers. Documents were examined to adapt interview questions so that information missing from the documents could be collected during the interview.

Key informants who processed pathogen genomic data and/or presented pathogen genomic results such as public health laboratory staff and genomic epidemiologists, as well as end users of pathogen genomic results such as frontline staff and public health staff, were invited to an interview or focus group via e-mail (31, 32). After providing their informed consent, key informants and end users participated in an interview, responding to questions relating to the case study, the outcomes of pathogen genomic results being used, the extent to which pathogen genomics informed public health responses against infectious disease, and the factors affecting the use and outcomes of pathogen genomics. Individual interviews were conducted for up to 60 min and focus group interviews up to 90 min. Interviews were conducted via Zoom or Teams, recorded in the videoconferencing platform and transcribed in the study SharePoint. The transcript was checked alongside the recording, and key informants were given the opportunity to review the transcript before it was used for data analysis. Once the key informant had confirmed the accuracy of the transcript, the recording was deleted in accordance with formal ethics approval to preserve confidentiality.

Key informant interviews and focus groups were conducted by four authors. JO conducted 15 interviews and 4 focus groups for 7 case studies. DW did 16 interviews for 2 case studies. AI and SP each did a case study involving an interview and two focus groups.

### 2.5 Within-case analysis

For each case study, documents and interview transcripts were uploaded to NVivo 14 (QSR International, Denver, Colorado), a software that supports qualitative data analysis. These documents and interview transcripts were coded according to a specific process (33). Briefly, this involved reading each document and interview transcript to become familiar with each information source, and annotating them to note any emergent reflections. An *a priori* list of codes was derived from the outcome and factor definition stage to code documents and interview transcripts into background information sections, outcomes and individual factors. Any new codes that emerged while reading a document or interview transcript was defined, added and applied to all other documents and interview transcripts across all case studies. The code groupings formed the basis for writing a case report summarizing the circumstances, outcome and factors of using pathogen genomic results in the case study.

### 2.6 Cross-case analysis

Case reports from different case studies were summarized in an Excel spreadsheet, with each column representing a case study and each row representing a background information section, outcome or factor. Case studies were compared to identify similarities and differences in context, outcome and factors which were noted in a separate column within the Excel spreadsheet. Outcomes among case studies were coded based on a coding framework that described the association between pathogen genomic results and public health investigations and responses against infectious disease (Table 2). These observations formed the basis for planning the results section to describe how pathogen genomics was used and implemented in public health. These results were contextualized with examples from individual case studies.

**Table 2:** Coding framework for determining outcomes of pathogen genomics use in public health.

Outcome code	Outcome
Information provider	Pathogen genomic results provided information about a clinical case to inform contact tracing
Response substantiation	Pathogen genomic results were used to confirm that an appropriate public health response had been enacted against the infectious disease
Change stimulus	Pathogen genomic results informed changes to public health responses and/or policies against infectious disease

## 3 Results

We examined 11 case studies of pathogen genomics use from different state and provincial public health systems across Australia and New Zealand. These case studies spanned different infectious diseases (Table 3), with 5 out of 11 case studies involving foodborne infectious diseases such as salmonellosis and yersiniosis and 3 out of 11 case studies involving tuberculosis. Other case studies focused on antimicrobial resistant (AMR) bacteria, melioidosis or shigellosis.

**Table 3:** The number of case studies by different infectious diseases.

Infectious disease	Number of case studies
Salmonellosis	4
Tuberculosis	3
Antimicrobial resistant bacteria	1
Melioidosis	1
Shigellosis	1
Yersiniosis	1

Most case studies (7 out of 11) described how pathogen genomics was used in outbreak responses against infectious disease. In contrast, the tuberculosis case studies used pathogen genomics to investigate clusters of cases. The case study about AMR bacteria was noteworthy in that pathogen genomics was used in both infectious disease surveillance and outbreak responses. While pathogen genomics was initially used in a research project to measure the prevalence of AMR bacteria in hospitals, it was later used to respond to hospital outbreaks of AMR bacteria.

### 3.1 The functions of pathogen genomics

Across the case studies, pathogen genomics served various functions in surveillance and outbreak investigations and responses against infectious disease.

First, pathogen genomics could include or exclude cases from a cluster based on their genomic links to the cluster. For two foodborne infectious disease case studies and one tuberculosis case study, this helped public health investigation teams prioritize clinical cases to interview or define the extent of the outbreak. Second, pathogen genomics could trace clusters back to an infection source. This was commonplace in foodborne infectious diseases where pathogen genomics identified similarities in genomic sequences between isolates from clinical cases and food products. This allowed public health investigation teams to link the outbreak to a food source, focusing their efforts. Third, pathogen genomics could provide more information on how the infectious disease was being transmitted. For example, one tuberculosis case study used pathogen genomic results to model how tuberculosis was being spread within a state. Fourth, pathogen genomics could provide new information on where a clinical case went and how they were infected. This may motivate the public health investigation team to re-interview a clinical case to collect information that they may have forgotten or withheld during the initial interview, as was the case in one tuberculosis case study. Lastly, pathogen genomics could detect AMR markers earlier compared to phenotypic resistance testing in laboratories. This allowed emerging AMR profiles to be identified rapidly so that public health responses can be initiated sooner.

### 3.2 Comparing outcomes across case studies

The impact that pathogen genomics had on public health investigations and responses against infectious disease varied among different case studies. For the tuberculosis case studies, it took time for enough *Mycobacterium tuberculosis* bacteria to be grown for sequencing. This meant genomic results were not available until after initial contact tracing of the clinical case was completed. Nevertheless, these genomic results could inform follow-up interviews with the clinical case to collect more information about how they were infected, identifying more contacts to test and interview. The genomic results could also be used to tailor clinical treatment to antimicrobial susceptibility results.

Similarly, pathogen genomic results could substantiate public health responses that had already started. In two case studies involving foodborne infectious diseases, epidemiological investigations had identified a potential source of infectious disease that may have caused the outbreak. This prompted the public health team to issue public health orders to close off the affected area prior to genomic results being available. Environmental samples were later collected and sequenced, where isolates from clinical and environmental samples had similar genomic sequences. This result corroborated the link between clinical cases and the source of infectious disease, providing confirmatory evidence that the appropriate public health response had been taken.

For other case studies, pathogen genomic results informed changes in public health responses and policies against infectious disease. For the AMR bacteria case study, pathogen genomics provided information on how the pathogen was introduced and transmitted, and how big the outbreak grew. Infection prevention and control teams used this information to enact strict measures that limited the spread of infectious disease and eliminated the source of the outbreak. Similarly, for the shigellosis case study, pathogen genomics was able to detect a multi-jurisdictional outbreak of multidrug-resistant *Shigella* which prompted public health alerts to be sent to healthcare professionals. Lastly, for the melioidosis case study, pathogen genomics identified a novel source of infectious disease that was added to the public health management guidelines for melioidosis. This enabled the public health team to work with the managers of an upcoming sporting event to manage the risk of melioidosis.

Pathogen genomics may also build relationships among different stakeholders. For example, in one salmonellosis case study, pathogen genomics identified genomic links between the clinical cases and the food source. This result convinced the food producers to allow environmental sampling of their production facilities and to implement interventions that reduced the pathogen load in their foods, leading to a reduction in cases. Similarly, the use of pathogen genomics in the yersiniosis case study convinced external stakeholders that pathogen genomics had an important role in guiding public health investigations against yersiniosis. Consequently, sequencing of *Yersinia* bacteria was now conducted twice weekly instead of *ad hoc*.

### 3.3 Comparing factors in phase I: Pre-analysis and analysis

#### 3.3.1 Trained staff

Three case studies had dedicated laboratory staff for sequencing and bioinformatics, while two case studies had laboratory staff who rotated between sequencing/bioinformatics and other laboratory tests. The number of available genomics-trained staff varied among different case studies. While one case study had enough genomics-trained staff to rotate around different parts of pathogen genomics, two case studies did not have enough genomics-trained staff to deliver genomic reports in a timely fashion. In one case study, a staff member was key in keeping the pathogen genomic program running. Should that staff member leave, it may have been more difficult to keep the program running.

#### 3.3.2 Data quality

None of the case studies had any issues with the quality of the genomic data. This was due to rigorous quality control processes and metrics being implemented to ensure that the genomic data was high in quality and coverage. Consequently, end users across all case studies felt confident in using genomic data to provide more information about the pathogen and to guide public health investigations and responses.

#### 3.3.3 Data linkage

Across the case studies, key informants highlighted the difficulties in linking genomic, epidemiological and laboratory data as they were commonly stored in separate databases. This was because it was impossible to store genomic data or regularly update clustering results in the notifications database at the time. Furthermore, the same sample could be coded under different identifiers across separate databases, necessitating the need to manually match identifiers from separate databases in an Excel spreadsheet. Additionally, surveillance systems in two case studies had difficulty updating genomic results such as clustering as additional genomic data were added to the system. The AMR bacteria case study, though, had started to set up semi-automatic processes to match genomic, epidemiological and laboratory data from different databases to integrate genomic data into their surveillance systems.

### 3.4 Comparing factors in phase II: Reporting and communication

#### 3.4.1 Reporting

End users such as epidemiologists received pathogen genomic reports containing information on individual cases, clusters, phylogenetic trees and/or drug resistance markers. These had been developed from technical genomic reports that, through consultations between laboratory staff and end users, had been simplified to only include key genomic results. Consequently, most end users found the genomic reports easy to understand and interpret. Reports were even easier to interpret when end users had a laboratory background on pathogen genomics, had been trained on pathogen genomics and/or could ask questions to the laboratory team on parts of the report they did not understand.

Pathogen genomic reports could also be further simplified into a summary that could be sent to end users who could not access or did not have time to read the report. These included decision-makers in public health teams who used the summaries to make public health decisions, and frontline staff such as doctors and nurses who used genomic results on tuberculosis to inform contact tracing and/or tailor treatment of clinical cases.

Besides pathogen genomic reports, visualization tools could also be developed to show end users how the pathogen was transmitted. For example, in the AMR bacteria case study, key informants developed two visualization tools that explained to end users how the outbreak started, how the pathogen spread and how transmission could be stopped.

#### 3.4.2 Collaboration

Staff from the public health laboratory worked closely with staff from the public health unit to incorporate pathogen genomic results into public health investigations and responses. This provided open communication lines to pass pathogen genomic results from the laboratory to the public health unit, as well as opportunities for pathogen genomic experts to meet end users to present the genomic results and plan further sequencing and bioinformatic analyses. The frequency of these meetings varied among case studies. For routine surveillance and outbreak responses of notifiable diseases such as tuberculosis and salmonellosis, pathogen genomic experts regularly met end users on a weekly, fortnightly or monthly basis. For other case studies where genomic surveillance of the infectious disease was irregular such as melioidosis, meetings were held on an *ad hoc* basis whenever genomic results were available.

#### 3.4.3 Timeliness

How long it took to generate pathogen genomic results may coincide with how pathogen genomics was used in different case studies. For the tuberculosis case studies, it took weeks to grow enough *M. tuberculosis* for sequencing, delaying the generation of genomic results. As genomic results were delivered after cases had completed the initial phase of contact tracing, they were more often used to increase knowledge about a case or cluster rather than to inform public health responses against tuberculosis. Turnaround times could also be prolonged by limitations on staffing and sequencing capacity, as well as writing long, complex genomic reports that, while containing valuable detail, could delay the delivery of key actionable insights.

Even if turnaround times to generate genomic results were reduced to 4 weeks, public health responses may have already been initiated beforehand. Hence, genomic results were more often used to confirm that the appropriate public health response had been taken. For instance, in the melioidosis case study, while clinical and environmental samples were sent for sequencing, epidemiological investigations identified and closed off the source of the outbreak. Genomic results received weeks later confirmed that closing off the source of the outbreak was the appropriate public health response.

In contrast, pathogen genomic results that were delivered in real time or near-real time (i.e., within a week) may more likely be used to inform public health responses. For instance, in the AMR bacteria case study, it took at most 7 days for the public health laboratory to receive and process the samples and generate a genomic report. Key informants reported that this rapid turnaround time allowed infection prevention and control teams to use the report to implement measures that controlled outbreaks against AMR bacteria.

### 3.5 Comparing factors in phase III: Implementation in public health practice

#### 3.5.1 Literacy

Across all case studies, the level of understanding of pathogen genomics varied among different stakeholders. Laboratory staff and managers who actively engaged in pathogen genomics were the most knowledgeable in the area. Most epidemiologists and public health staff also had enough knowledge in pathogen genomics to interpret and understand the genomic reports. Those who had previously conducted pathogen genomics in the laboratory could use their expertise to interpret and understand the genomic reports by themselves. Otherwise, public health staff were reliant on the laboratory’s summary of the genomic results to understand them. In contrast, frontline staff such as doctors and nurses only had a basic, if any, understanding of pathogen genomics and how it could be used in their clinical practice. Consequently, they were less likely to be engaged in or use pathogen genomics in their work, as well as receive and read genomic reports.

An additional challenge for frontline staff was the high staff turnover which may produce variability in their level of understanding towards pathogen genomics. In response to this, three case studies delivered pathogen genomics training to end users, particularly frontline staff. Training programs were associated with enhanced end users’ understanding of pathogen genomics. This may lead to increased utilization of pathogen genomic services and improved ability to interpret and use pathogen genomic results to guide public health investigations and responses and clinical treatment.

#### 3.5.2 Attitude

Across the case studies, different end users held contrasting views towards pathogen genomics. On the one hand, public health staff and their managers felt positive about pathogen genomics, and were willing to use it in infectious disease surveillance and outbreak responses. This positive attitude was strengthened if pathogen genomics was previously used to investigate infectious disease outbreaks. On the other hand, frontline staff such as doctors and nurses were less likely to feel positive about pathogen genomics. In the tuberculosis case studies, frontline staff spent more time on contact tracing and treatment of clinical cases than interpreting genomic results.

Training programs on pathogen genomics had been shown to improve end users’ knowledge and attitudes towards pathogen genomics. For example, end users of a salmonellosis case study initially felt hesitant about transitioning to pathogen genomics. However, after being trained on pathogen genomics, they were more receptive to using genomic results to investigate infectious disease outbreaks. Similarly, for the AMR bacteria case study, training frontline staff on pathogen genomics led to them feeling positive about pathogen genomics, and increased their willingness to learn more about pathogen genomic services being offered in the jurisdiction.

#### 3.5.3 Novelty

In a salmonellosis case study and the melioidosis case study, pathogen genomics provided conclusive evidence of what laboratory tests and epidemiological investigations had already found. In these case studies, initial public health investigations identified a potential source of the outbreak. Pathogen genomic results received a few weeks later found a genomic link between the clinical cases and the environmental source, providing conclusive evidence to confirm the source of the outbreak.

In other case studies, pathogen genomics provided new information about a cluster or outbreak of an infectious disease that neither laboratory tests nor epidemiological investigations had previously found. In particular, the higher resolution of pathogen genomics may enable clinical cases to be sorted into smaller clusters which may have enabled outbreaks to be detected sooner before they became evident from traditional laboratory tests or epidemiological surveillance. For instance, in the AMR bacteria case study, pathogen genomics was instrumental in linking clinical cases to a transmission chain or existing cluster and tracing them back to an environmental source. This gave infection prevention and control teams the evidence they needed to target the environmental source, ending several outbreaks. Pathogen genomics was also used in a salmonellosis case study to highlight genomic links between isolates from clinical cases, the food source and food production facilities, resulting in them clustering together. This provided the public health team with conclusive proof that the outbreak could be traced back to the food production facilities, allowing them to convince food producers of the scale of the problem and the need to initiate environmental sampling to gather the necessary data to inform the required interventions. This resulted in a reduction of *Salmonella* prevalence in their food.

### 3.6 Finances

Across the case studies and the three PG-PHASE phases, securing sustainable government funding to run pathogen genomic services was a challenge. While one case study received some government funding to establish and maintain the pathogen genomics program, government funding was either limited or not sustained in most case studies. Insufficient government funding and/or laboratory capacity forced public health teams to decide which samples to sequence. For instance, in the melioidosis case study, limits on the number of samples that could be sequenced forced the public health team to select environmental samples that were hypothesized to be potential sources of the outbreak. Conversely, if government funding was not sustained or non-existent, pathogen genomic services operated on a fee-for-service basis, where hospitals and health services had to pay to have their samples sequenced. Given the high costs of sequencing samples, hospitals and health services only utilized pathogen genomic services whenever they needed information on how to treat their patients. This limited the number of clinical samples that could be sequenced to inform public health investigations and responses.

## 4 Discussion

**Figure 2:**
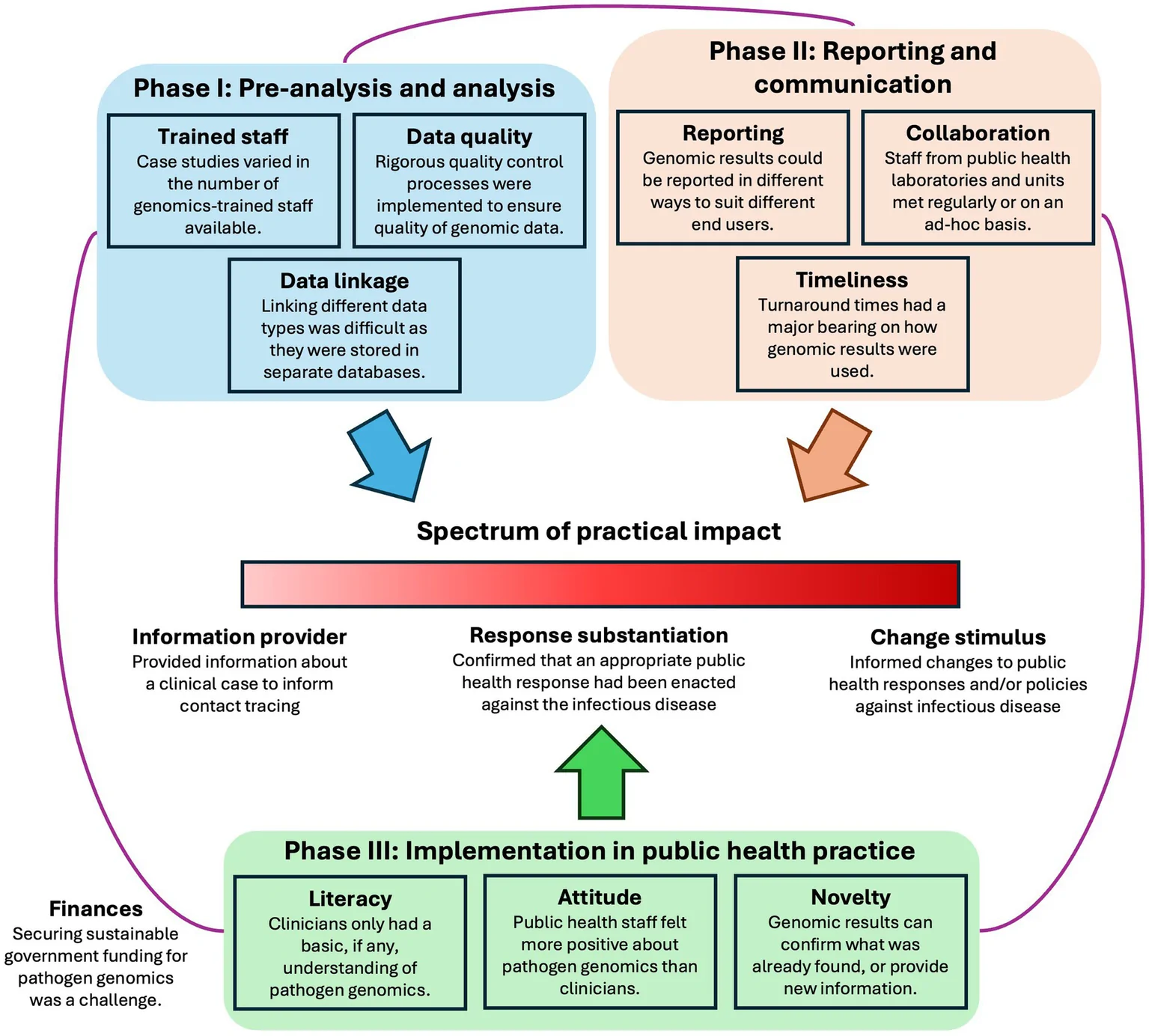
Summary of study results.

Countries globally are incorporating pathogen genomics into their public health systems to augment prospective surveillance and outbreak responses against infectious disease. Despite this, the implementation and outcomes of this process are rarely studied. In particular, it is unclear how end users are using pathogen genomic results to inform public health surveillance and responses against infectious disease. This study examined case studies across Australia and New Zealand to describe the processes and outcomes of using pathogen genomics in public health to guide surveillance and outbreak responses against infectious disease. Examining case studies allowed us to describe the myriad functions of pathogen genomics in public health, the varied levels of impact pathogen genomics had on surveillance and outbreak responses against infectious disease, and how individual factors may facilitate the implementation, use and outcomes of pathogen genomics in public health (Figure 2). Together, these findings fill a research gap in providing current accounts of how pathogen genomics is implemented and used in public health, providing lessons to improve the implementation, use and outcomes of public health pathogen genomics programs globally.

### 4.1 Exploring different outcomes of pathogen genomics

Among the case studies, we found differences in the impact pathogen genomics had on public health investigations and responses against infectious disease. In most case studies, pathogen genomics was used to provide more information about a case or cluster or to support public health responses that had already started or occurred. In a small number of case studies, pathogen genomic results were linked to changes in public health responses and policies against infectious disease and establishing relationships among different stakeholders. These results conflict with the predominant assumption that the use of pathogen genomic results must always lead to changes in public health action against infectious disease (34, 35). However, they align with the findings of a Delphi study (36) where experts agreed that pathogen genomics could be used not only to support outbreak risk assessment and management, but also to monitor the emergence of new variants. Our results suggest a spectrum of practical impacts of pathogen genomics on public health, even if genomic data do not always directly influence responses against infectious disease. This spectrum aligns with the many roles pathogen genomics played in the COVID-19 pandemic. Pathogen genomics was used not only as an investigative tool to examine individual clusters (37) or measure the incidence of specific SARS-CoV-2 variants (38), but it was also used to inform changes in infection prevention and control policies (39, 40) and implementation of public health responses against COVID-19 (17).

The potential impact of pathogen genomics on public health may depend on the context and constraints of the case study, as well as the timeliness of receiving genomic results and the novelty of genomic results delivering new information compared to other data types. Looking at timeliness, genomic results appeared to be less likely used to inform public health responses if they were delivered 4 weeks or more after sending samples for sequencing. Conversely, genomic results delivered within a week of sending samples for sequencing appeared to more likely inform public health investigations and responses against infectious disease. These results corroborate several studies that highlighted the importance of short turnaround times (of 1 week or less) in supporting outbreak detection and response (2, 4, 41). For reference, turnaround times for sequencing of most SARS-CoV-2 samples during the COVID-19 pandemic were up to 14 days (37, 38), though a rapid turnaround time of 1.5 days within a Queensland hospital was found to enable many infection prevention and control initiatives to be rapidly implemented (13). Turnaround times could be reduced by optimizing processes within the pathogen genomics workflow, including conducting sequencing in-house (2), streamlining sequencing and data analysis processes (42), decentralizing sequencing to multiple laboratories (43) and directly sequencing clinical samples via metagenomics (44). Finding ways to reduce turnaround times may allow end users to receive and use genomic results sooner to inform public health investigations and responses against infectious disease.

For novelty, pathogen genomic results were likely to influence public health investigations and responses if they provided new information about a cluster or outbreak. In some case studies, pathogen genomic results confirmed what laboratory tests and epidemiological investigations had previously found. In other case studies, pathogen genomics provided new information about infectious disease transmission not previously found by laboratory tests or epidemiological investigations. This occurred when genomic results could be turned around quickly (as in the AMR bacteria case study) or when conclusive proof of the transmission or source of infectious disease was required (as in a salmonellosis case study). These results support a recent Delphi survey where genomics experts agreed that pathogens should be prioritized for sequencing based on whether genomic results could inform public health action (45).

The results of these two factors may highlight that the contribution of pathogen genomics in public health could be maximized when (1) results could be turned around quickly or (2) results provided new information about the surveillance or outbreak of a pathogen. Hence, end users and decision makers of pathogen genomics could discuss which pathogens should undergo sequencing, how often they should be sequenced, and how genomic results should be used. This might be particularly true when resources are limited such as in low-and-middle-income countries, where pathogen genomics needs to be piloted in a small number of pathogens for specific purposes before it is scaled up (46).

Regardless of context, there were some consistent factors across case studies that could support the implementation of pathogen genomics in public health. A few noteworthy factors are discussed below.

### 4.2 Data quality and data linkage

The quality and linkage of genomic data may facilitate the use of genomic results among end users. High quality data in pathogen genomics is important to avoid misinterpreting genomic results and acting on false conclusions (42, 47). Among the case studies, we did not find any data quality issues due to quality control processes being implemented, allowing end users to feel confident in the results. These results concur with previous studies highlighting the importance of quality control processes in producing accurate results that could inform public health investigations and responses against infectious disease (21, 42, 48). High quality data, consistently formatted, may also enable genomic data to be linked to clinical and epidemiological data (49).

It is known that genomic results should be interpreted alongside epidemiological results to strengthen conclusions on links between cases and infection sources (1, 36, 48). The advantages of linking genomic, laboratory and epidemiological data was seen during the COVID-19 pandemic as it enhanced understanding of SARS-CoV-2 transmission (17, 37). Among case studies, however, linking genomic and epidemiological data was difficult as they were stored in separate databases, requiring them to be manually matched. These experiences corroborate those of the United States and Canada where genomic data were stored in specialized databases and bioinformatics systems, away from databases that stored clinical, laboratory and epidemiological data (50, 51). Placing different types of data into separate databases addresses data security and privacy concerns that are high priorities for policymakers and the community (22, 52, 53). At the same time, this may make it difficult to link genomic and epidemiological data which could limit the insights that can be drawn (51, 54). Hence, there is a need to balance the protection of individual privacy with the public health benefits from linking genomic data to other data types. Fortunately, privacy-preserving record linkage techniques exist where different data types can be linked without needing individuals’ personal information (55). These techniques may help protect data security and individual privacy while linking genomic data to other data types to draw better insights about infectious disease transmission.

### 4.3 Reporting

End users found genomic reports easy to understand and use. However, access to and complexity of genomic reports varied among end users. The genomic reports that epidemiologists received contained a subset of results from technical genomic reports generated by public health laboratories. These genomic reports could be further simplified into summaries for decision-makers and frontline staff who may not have access or time to read the reports. These results suggest that access to genomic results could affect whether and how end users utilize them. One way to improve pathogen genomics use might be to make pathogen genomic results more accessible to end users via different presentation formats. Previous studies have developed standardized genomic reports that are formatted to suit different end users (29, 56). For instance, genomic results could be presented as a one-page summary report for frontline staff, or a longer report for public health laboratories and researchers (30).

Alternatively, visualization tools, as explored in the AMR bacteria case study, could be another avenue to make genomic results more accessible to end users, particularly those with low genomics literacy such as policymakers (57). These tools could describe the distribution and spread of infectious disease variants. Together, the use of different reporting formats to present genomic results could enhance its accessibility to end users which may lead to increased utilization rates.

### 4.4 Literacy and attitudes

Across the case studies, stakeholders in public health had varying levels of knowledge and attitudes towards pathogen genomics. Public health laboratory and unit staff had moderate to high levels of knowledge in pathogen genomics, and generally felt positive about the technology. Consequently, they were able to interpret and understand genomic reports, and were willing to use pathogen genomics in public health responses against infectious disease. In contrast, frontline staff were less likely to have any knowledge of, or to have a positive attitude towards, pathogen genomics. Consequently, they were less likely to work with genomic results in their role. These results are similar to one study (6) which found that the capacity to understand and interpret genomic results was the largest barrier to acceptance towards pathogen genomics. Another study found that stakeholders with high genomics literacy such as clinical biologists and bioinformaticians were more likely to have a positive attitude towards pathogen genomics and to use genomic results (23). In contrast, not many frontline staff were trained on pathogen genomics which was associated with reduced enthusiasm towards pathogen genomics and lower rates of use. The authors concluded that training was needed to increase genomics literacy among public health staff and frontline staff. This is something we have found in our case studies, as training programs were linked to enhanced end users’ understanding of pathogen genomics and improved attitudes towards and utilization of pathogen genomic services.

Collectively, genomics literacy might be a key factor facilitating the use and outcomes of pathogen genomics in public health. This may underline the need to train all public health staff on pathogen genomics. In particular, given the high staff turnover in health facilities, pathogen genomics could be incorporated into frontline staff training curricula so that frontline staff already have knowledge in pathogen genomics when they work in health facilities.

### 4.5 Limitations and strengths

There were some limitations in the study. Firstly, all 11 case studies used pathogen genomic results to some degree. Including at least one case study where pathogen genomic results were available but not utilized by end users may have provided more information on the factors driving the non-use of genomic data. Secondly, for most case studies, we could not access internal documents from public health units and laboratories due to the need to protect the privacy of individuals (58). In these case studies, information was drawn purely from publicly available documents to develop the interview questions required to collect the missing information. Lastly, we could not interview any policymakers who made executive decisions on public health responses, so we could not draw many conclusions on how pathogen genomics affected high-level health policy.

Despite these limitations, there were some strengths. Firstly, we employed a study design that enabled a detailed investigation of how pathogen genomics was used across case studies. This allowed us to preserve the context of the case studies to describe how pathogen genomics was used and the factors that influence this use. Secondly, we managed to collect case studies across a number of infectious diseases and jurisdictions across Australia and New Zealand. This enabled us to study various contexts where pathogen genomics was used, further elevating the level of detail in the results. Lastly, we were able to triangulate findings across documents and interviews. The documents provided the background behind the case studies, enabling us to tailor interview questions to collect new information on the case studies from key informants.

## 5 Conclusion

Our study describes the various functions of pathogen genomics in public health, the different impacts that arise from this use, and how pathogen genomics is implemented in public health. These results fill a research gap in terms of understanding the realities of implementing and using pathogen genomics in public health. Three implications arise from this study. Firstly, the results guide future implementation research in public health pathogen genomics such as comparing implementation and outcomes across countries, or assessing the interoperability of different data types. Secondly, these results can inform the development of tools that countries can use to assess capacity across different parts of pathogen genomics and identify areas of improvement. Lastly, the results highlight actions countries could take to improve public health pathogen genomics programs, such as running training programs on pathogen genomics, building data systems to more seamlessly link different data types and finding efficiencies to reduce turnaround times for end users to receive genomic results. These implications may improve the implementation and use of pathogen genomics in public health, enhancing its contribution to more tailored, timely and effective responses against infectious disease.

## Australian pathogen genomics program partners

For information on AusPathoGen, please visit https://www.auspathogen.org.au/.

## Data Availability

The datasets presented in this article are not readily available because data generated and analyzed in the current study are not publicly available due to the confidential nature of individual case studies and the need to preserve the anonymity of key informants and key contacts.
